# Synthetic or Natural (Bio-Based) Hydroxyapatite? A Systematic Comparison between Biomimetic Nanostructured Coatings Produced by Ionized Jet Deposition

**DOI:** 10.3390/nano14161332

**Published:** 2024-08-09

**Authors:** Matteo Montesissa, Enrico Sassoni, Marco Boi, Giorgia Borciani, Elisa Boanini, Gabriela Graziani

**Affiliations:** 1 BST Biomedical Science and Technologies and Nanobiotechnology Lab, IRCCS Istituto Ortopedico Rizzoli, 40136 Bologna, Italy; marco.boi@ior.it (M.B.); giorgia.borciani@ior.it (G.B.); 2 Department of Civil, Chemical, Environmental and Materials Engineering, University of Bologna, 40131 Bologna, Italy; enrico.sassoni2@unibo.it; 3 Department of Chemistry “Giacomo Ciamician”, University of Bologna, 40126 Bologna, Italy; elisa.boanini@unibo.it

**Keywords:** nanostructured coatings, spine surgery, calcium phosphates, ionized jet deposition, bone regeneration

## Abstract

Calcium phosphate (CaP)-based materials are largely explored in orthopedics, to increase osseointegration of the prostheses and specifically in spine surgery, to permit better fusion. To address these aims, nanostructured biogenic apatite coatings are emerging, since they better mimic the characteristics of the host tissue, thus potentially being better candidates compared to their synthetic counterpart. Here, we compare hydroxyapatite (HA) nanostructured coatings, obtained by ionized jet deposition, starting from synthetic and natural sources. The starting materials and the corresponding films are characterized and compared from a compositional and morphological point of view, then their stability is studied after post-treatment annealing. Although all the films are formed by globular aggregates and show morphological features at different scales (from nano to micro), significant differences are found in composition between the synthetic and naturally derived HA in terms of magnesium and sodium content, carbonate substitution and Ca/P ratio, while differences between the coatings obtained by the different natural HA sources are minor. In addition, the shape of the aggregates is also target-dependent. All coatings have a good stability after over 14 days of immersion in medium, with natural apatite coatings showing a better behavior, as no cracking and detachments are observed during immersion. Based on these results, both synthetic and naturally derived apatitic materials appear promising for applications in spine surgery, with coatings from natural sources possessing physiochemical properties more similar to the mineral phase of the human bone tissue.

## 1. Introduction

Calcium phosphate (CaP)-based materials are widely studied to improve the performance of medical devices for orthopedics and dentistry. In orthopedics, the mobilization (aseptic loosening) and the formation of a fibrous tissues capsule between the implant surface and the surrounding bone that prevent correct osseointegration are among the main causes of implant failure [1]. CaPs are used to overcome this problem and obtain a firm bone bonding between the implant and native bone through osseointegration, because of their biocompatibility and bioactivity [2,3,4,5]. Indeed, calcium phosphates represent the main component in human bone, which is composed of around 70% biogenic hydroxyapatite [3,6,7]. CaP-based materials are also used in the specific field of spine surgery, to permit fusion between adjacent vertebrae [3]. To meet these goals, CaP-based materials can be used in different forms, such as nanoparticles, scaffolds, bone cements and coatings. In particular, the latter are widely diffused for the functionalization of implants [2,8]. Coatings, in particular, represent an interesting solution to increase the biomimetism of the implants, because they can be applied on the surface of bio-inert metallic bone prostheses to favor host cellular adhesion, proliferation and differentiation and thus the formation of new bone in vivo [4,9].

Nanostructured thin CaP-based coatings are promising, since submicrometric thickness allows the avoidance of the mechanical mismatch that leads to cracking, while nanostructuring favors hosts cell adhesion and proliferation and can even provide cues for faster osteogenic differentiation [9,10,11,12,13]. In particular, it is demonstrated that the use of calcium phosphate-based materials for coating fabrication in spine surgery can improve their osseointegration capability. Intervertebral implants, cages and screws coated with hydroxyapatite improve the new bone formation on their surface, increasing the fixation strength and avoiding aseptic loosening [14,15].

In the literature, different fabrication techniques are presented to obtain the coatings, but plasma-assisted and Physical Vacuum Deposition (PVD) techniques are the most used, as is the case at an industrial level [16]. Currently, plasma spray (PS), is the most commercially diffused deposition system for calcium phosphate coatings [17,18,19] and hydroxyapatite (HA) is the most used calcium phosphate for biomimetic film fabrication, due to its similarity with the mineral phase of bone tissue.

However, due to their relatively high thickness (50–200 µm [17,18,19]), PS films can show some drawbacks, including scarce uniformity or adhesion to the substrate surface, causing delamination and cracking [19,20,21] or the formation of decomposition phases upon deposition [22].

To overcome the problem connected with PS, other plasma-assisted techniques have been proposed to manufacture HA coatings of submicrometric thickness, including magnetron sputtering (MS), pulsed laser deposition (PLD), pulsed electron deposition (PED) and ionized jet deposition (IJD) [19,20,23,24]. PED consists of deposition of the starting target material through the ablation of a pulsed electron beam [25]. IJD is an evolution of the PED technique, where the source of the electron beam is the change from a dielectric tube to a gas jet directed into a solid target through a system of trigger and auxiliary electrodes [25]. In IJD, as in PED, the starting target material is ablated by a high frequency and energy pulsed electron beam, but in IJD the conservation of stoichiometry is higher and the characteristics of the coating also show some differences [26]. Our work on PED [27] and on IJD [28] has demonstrated that they allow one to obtain a nanostructured coating with a high fidelity in the stoichiometry transfer from the deposition target to the coatings, and also for complex materials, and that CaP-based IJD coatings have high bioactivity, thanks to their peculiar morphological and compositional characteristics [13,29,30].

Apart from the morphological characteristics of the coatings, which derive from the deposition technique, another limitation connected to traditional coatings is that biogenic apatite differs from the stoichiometric one in terms of composition (presence of carbonates and trace ions), crystallinity and solubility, and, overall, of the type and amount of ions released in the peri-implant environment. In the literature, most of the studies on plasma-assisted techniques focus on developing HA coatings starting from stoichiometric hydroxyapatite targets. Synthetic HA has a Ca/P ratio of 1.67 [31], which differs from the biological one (Ca/P ratio around 1.64). In fact, biological HA is a hydroxyl-deficient and carbonate-rich apatite [7] characterized by the presence of different ion substitutions such as Sr^2+^, Ba^2+^, Mg^2+^, Na^+^, K^+^ and F^−^ e Cl^−^, that alter the Ca/P molar ratio and increase the solubility with respect to synthetic HA [32] (Table 1).

New HA-based materials from waste and animal sources are being increasingly investigated [18,33,34,35]. Indeed, bone-like materials from animal waste, marine shells and fish bones can represent promising naturally derived HA sources [36,37] due to their similarity in composition with human bone hydroxyapatite (Table 1). In particular, mammalian bone represents one of the waste products from the food industry, so it is easy to collect from butcher shops or as a byproduct of the food industry and it does not involve high costs and ethical issues, and instead appears as a promising green material [32].

There are some advantages in using naturally derived and biogenic HA compared to synthetic HA; first, the use of green materials is to be preferred to synthetic counterparts [36]. In addition, biogenic HA has a composition that is more similar to the human bone (Table 1), in terms of solubility, crystallinity and trace elements [36,37]. The presence of these trace elements plays a very important role for bone metabolism; in fact, they accelerate and stimulate bone regeneration and formation [21,37,38].

Our studies [39] have demonstrated that bones from different animal sources show differences in composition in terms of the presence of trace ions, and that bovine bone can be deposited by IJD to obtain nanostructured biomimetic coatings [13,28], showing tunable characteristics and high biomimicry and bioactivity, as they can boost MSC viability and differentiation. Finally, we have preliminarily demonstrated that IJD coatings can be obtained by the deposition of apatite from different animal sources [39].

In this work, we have produced HA-based nanostructured coating by ionized jet deposition (IJD). We systematically compare the compositional and morphological characteristics of coatings obtained by stoichiometric hydroxyapatite with those obtained by animal-derived apatite, as well as the differences that exist between coatings derived from different animals. Finally, we show how heating modifies the characteristics of animal-derived and stoichiometric apatite coatings, enhancing their crystallinity.

## 2. Materials and Methods

### 2.1. Materials

Synthetic HA targets (S_HA_t) for IJD are realized starting from powders prepared using Ca(NO_3_)_2_·4H_2_O, NH_4_OH and (NH_4_)_2_HPO_4_ (Carlo Erba, Milano, Italy).

HA nanocrystals are synthetized under nitrogen atmosphere and using CO_2_-free distilled water. A solution of 50 mL of 1.08 M Ca(NO_3_)_2_·4H_2_O (pH adjusted to 10 adding NH_4_OH) is heated up to 90 °C and 50 mL of 0.65 M (NH_4_)_2_HPO_4_ solution (pH 10 adjusted with NH_4_OH) are added dropwise under stirring. The resulting suspension is stirred for 5 h at 90 °C; afterwards the precipitate is isolated by centrifugation at 10,000 rpm, repeatedly washed with CO_2_-free distilled water and dried at 37 °C. An amount of 4.75 g HA powder is pressed with 0.25 g of starch into cylindrical molds (Ø = 2.8 cm) to manufacture the synthetic HA targets (S_HA_t) and obtain cylindrical samples for IJD.

Biogenic HA targets are obtained from different animal bone sources. In detail, bovine, equine and porcine cortical bone are collected from local butcher shops. They are deproteinized for 14 days through immersion, under continuous stirring, in a solution of 2.7% NaClO and rinsing in distilled water. Finally, bones shafts are cut in shapes suitable for the IJD (bovine targets B_HA_t, equine targets E_HA_t and porcine targets P_HA_t).

Deposition is carried out on medical grade titanium alloy disks (Grade 23 Ti6Al4V ELI alloy, diameter 5mm, height 3mm, superficial roughness Ra 5 μm, Citieffe S.r.l., Bologna, Italy). Glass and silicon wafers (p-type doped monocrystalline (100) native silicon, size 5 × 5 mm, thickness 1 mm, Fondazione Bruno Kessler, Trento, Italy) are also used as a reference. All the substrates are cleaned with ultrasonic agitation in a 50:50 ethanol and isopropyl alcohol solution for 15 min and dried under nitrogen flow.

### 2.2. Methods

#### 2.2.1. Targets Characterization

The composition of all targets (S_HA_t, B_HA_t, E_HA_t and P_HA_t) is analyzed by Fourier Transform InfraRed (FT-IR) spectroscopy in ATR mode (Perkin Elmer Spectrum two, diamond crystal, resolution 4 cm^−1^, 16 scans, data interval 1 cm^−1^, Waltham, MA, USA) and by an Energy Dispersive X-ray Spectrometry system (EDS, Oxford Instruments equipped with a SDD detector, Abingdon-on-Thames, UK).

#### 2.2.2. IJD

Biomimetic nanostructured coatings are all obtained by IJD (Noivion Srl, Rovereto (TN), Italy), starting with stoichiometric HA (S_HA_c), bovine bone (B_HA_c), equine bone (E_HA_c) and porcine bone (P_HA_c). In this case, all the materials used are ceramics or ceramic-like, so the deposition parameters and setup are the same for all targets [29]. In particular, the working voltage is set to 18 kV and the electron beam frequency to 7Hz; the targets are mounted on a rotating target holder and placed at a distance of 8 cm from the substrate surfaces. The targets are ablated through an electron beam (fast pulse of 100 ns with high energy and density of 10 J and 109 W cm^−2^, respectively). The deposition process is performed under high vacuum conditions and in oxygen atmosphere (chamber pressure inside around 2 × 10^−4^ mbar) and at room temperature.

All the substrates are maintained in rotation during the whole process to ensure uniformity in the coating. The deposition time is set to 30 min to avoid cracks and delamination, based on previous results.

To optimize crystallinity, part of the coatings are heated at 400 °C for 1 h after deposition.

#### 2.2.3. Morphological Characterization of the Films

HA films are analyzed from a morphological point of view by a Field Emission Gun Scanning Electron Microscope (FEG-SEM, Tescan Mira3, CZ, working distance 10 mm, voltage 10 kV) with the aim of understanding if the morphology and texture of the coatings may be influenced by the composition of the targets. The FEG-SEM images obtained at a higher magnification (50,000×) are also used to define the aggregate dimension by ImageJ software (1.48 v., National Institutes of Health, Bethesda, MD, USA). For each coating, maximum (DM), minimum (Dm) and average (Da) diameters are calculated, and the distribution of aggregate dimensions is studied. To define their distribution, the aggregates are divided in five different diameter groups: d < 100 nm, 100 nm ≤ d < 250 nm, 250 nm ≤ d < 500 nm, 500 nm ≤ d < 1000 nm and d ≥ 1000 nm. The percentage of aggregates having diameters within each range is calculated. To measure the thickness of coatings realized at room temperature and with post-deposition annealing treatment at 400 °C, a scratch is made with a scalpel on the coating surface and images are acquired through FEG-SEM at 5000× magnification. The are 6 different measurements made on every coating with ImageJ software and then averaged.

#### 2.2.4. Physiochemical Characterization of the Films

Coating composition is evaluated using FT-IR and EDS. EDS analysis is performed with the same instrument described above. In particular, the Ca/P molar ratio and ion element trace are studied and compared with the values of the corresponding starting targets. FT-IR spectra are acquired in ATR mode at a resolution of 4 cm^−1^, with 16 scans and a data interval of 1 cm^−1^. Also, in this case, the resulting spectra are compared with those of the targets materials.

#### 2.2.5. The Effect of Thermal Treatments

The possible effects on the coating morphology, in terms of changes in aggregate shape, cracks or delamination formation, are analyzed by FEG-SEM observation of S_HA_c and B_HA_c (5000× magnification). In terms of composition, the film analysis is performed by FT-IR. The capability of IJD to preserve the starting composition of the target materials in the coatings as well as the effect of temperature is studied.

#### 2.2.6. Stability Profile

Stability profile has been studied for synthetic (S_HA_c) and bovine-derived HA (B_HA_c) coatings after post-deposition annealing. To do so, dissolution tests are carried out on coated titanium alloy cylinders. Before the tests, the samples are sterilized for 1 h under UV exposure. After the sterilization process, the samples are immersed in 24 well plates, in 2 mL of Minimum Essential Medium Eagle Alpha Modification (α-MEM) and incubated at 37 °C with 95% humidity. The samples are kept in stationary condition in medium for different experimental times: 1, 7 and 14 days. At every timepoint, the samples are collected, washed in distilled water, and observed by FEG-SEM to study the residual presence of the coatings and the possible formation of defects.

## 3. Results

### 3.1. Target Characterization

**Figure 1:**
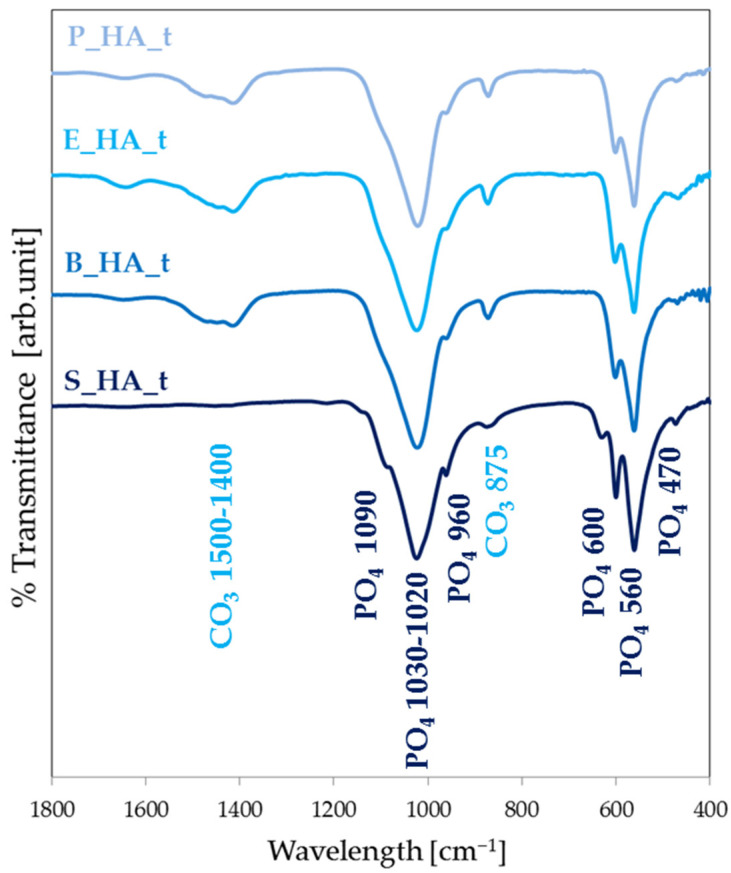
FT-IR spectra of the targets for differently sourced HA.

The composition of the targets is analyzed by FT-IR and the resulting spectra are in Figure 1. All targets show the characteristic bands of hydroxyapatite. In particular, they possess bands at 1090 cm^−1^ (antisymmetric stretch ν_3_PO_4_), 1030–1020 cm^−1^ (antisymmetric stretch ν_3_PO_4_), 960 cm^−1^ (ν_1_PO_4_ symmetric stretch), 600 and 560 cm^−1^ (antisymmetric bend ν_4_PO_4_) and 470 cm^−1^ (ν_4_PO_4_ bend) [29,39,40,41,42,43].

In addition, samples of naturally derived HA also show bands of carbonates in the 1500–1400 cm^−1^ area (asymmetrical and symmetrical stretching modes of CO_3_ν_3_) and at 875 cm^−1^ (ν_2_CO_3_) [29,39,40,41,42,43]. This behavior represents one of the major differences between synthetic HA and biological HA, which results in a more carbonate-rich apatitic phase [7].

The absence of bands characteristic of collagen in the 1400–1600 cm^−1^ range confirms the effectiveness of the deproteinization process [28].

Furthermore, in the spectrum of S_HA_t, the bands are sharper and the band at 630 cm^−1^, due to the ordered presence of the OH group, is clearly appreciable, while absent in all the other spectra. This suggests a higher crystallinity of S_HA_t compared to all the other materials.

Differences among the various target materials can also be seen from the EDS analyses (Table 2). In particular, Na and Mg are absent in the synthetic HA, as expected, but some differences can also be observed in their quantities among bone from different animals. Indeed, bovine has the higher amount of Na and Mg ion with respect to the other two naturally derived bone sources (equine and porcine). Sodium and magnesium are involved in human bone metabolic activity and growth [44] and are the most common substitutions for calcium in natural hydroxyapatite [32], so a higher presence is expected to have a positive impact on the bioactivity of the material. In terms of the Ca/P ratio, the value for stoichiometric apatite is 1.67. All biological HAs possess lower Ca/P ratios with respect to the stoichiometric value, indicating a lower degree of order in the solid materials. In fact, the presence of different ion substitutions in the HA lattice alters and reduces the value of the Ca/P ratio in biogenic HA [31,32].

### 3.2. Coatings Morphology

**Figure 2:**
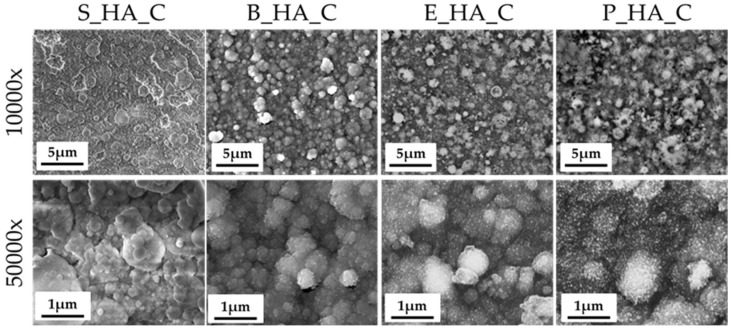
FEG-SEM images of the different HA sources’ coatings.

**Figure 3:**
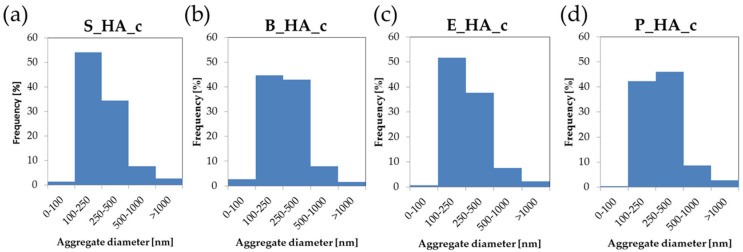
Globular aggregate distribution for the four different coatings ((**a**) synthetic HA, (**b**) bovine HA, (**c**) equine HA and (**d**) porcine HA). The aggregate diameters are divided in five different dimensions: d < 100 nm, 100 nm ≤ d < 250 nm, 250 nm ≤ d < 500 nm, 500 nm ≤ d < 1000 nm and d ≥ 1000 nm.

The morphology and nanostructure of the films is shown in Figure 2. Images at lower magnification (Figure 2, top) show that all coatings, not including the deposition target, are homogeneous and uniformly distributed on the substrate surface, as underlined by the absence of uncoated areas and/or surface defects (cracks, delamination). At higher magnification (50,000×), we can observe that all films are nanostructured and composed by globular aggregates with different dimensions. In fact, they are formed by aggregates having diameters from around 80 nm up to 1500 nm (Table 3). In general, for all different sources, the average aggregate diameters are around 300 nm (Table 3 and Figure 3). The aggregates in S_HA_c show a more irregular morphology with respect to the naturally derived HA coatings, which have more spherical grains. Some minor differences are also observed among coatings obtained by different bone sources, with bovine bone being characterized by smaller grains (in the range below 100 nm) and showing the most uniform distribution of diameters. Porcine bone, instead, is generally composed by larger grains, with a significant fraction being above 1 micron. This indicates that, up to a certain extent, it is possible to tune the morphological characteristics of the coatings by selecting different targets.

The thickness for coatings manufactured at room temperature and treated by post-deposition annealing treatment at 400 °C are reported in Table 4. In general, no significant differences are observed between the coatings obtained from the different HA sources, all showing a thickness around 100–120 nm. As expected, heating does not modify this parameter either.

### 3.3. Physiochemical Coatings Characterization

**Figure 4:**
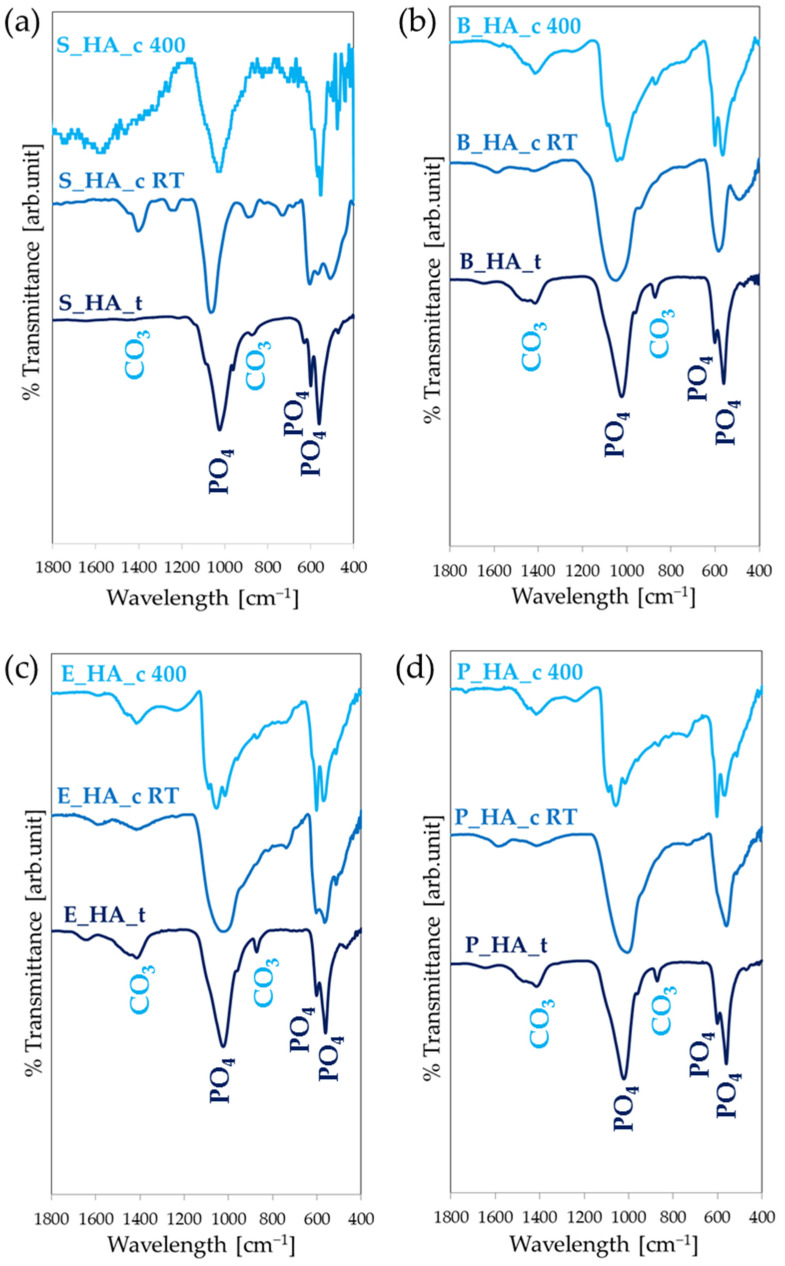
FT-IR spectra of different source coatings. Coatings are shown, starting from synthetic HA (**a**), bovine HA (**b**), equine HA (**c**) and porcine HA (**d**).

The deposition of synthetic HA (Figure 4a) results in the formation of weakly crystalline coatings, showing broad bands compatible with calcium phosphates around 1100–1000 cm^−1^, 600 and 560 cm^−1^. During the deposition treatment, the formation of carbonates is observed (bands at 1400 and 870 cm^−1^). In general, the spectrum of synthetic HA (S_HA_c RT) indicates that the high crystallinity of the starting HA powder is lost. In fact, the main stretching band of PO_4_ appears broad and poorly defined, compared to that of the target. Indeed, the bands at 1090 and 960 cm^−1^ cannot be distinguished and the two bands at 600 and 560 cm^−1^ are less scarcely evident. Films obtained by different naturally derived HA have similar spectra (Figure 4b–d). Following heat treatment, the crystallinity slightly increases, as suggested by the fact that the bands at 1000–1100 cm^−1^ appear more defined and the bands at 1090 and 960 cm^−1^ start being visible. In addition, band characteristics of carbonated HA appear at 1500–1400 cm^−1^.

The magnesium and sodium ion presence in the coatings are studied through EDS. From Table 1, it is possible to notice a correspondence between the values recorded for the coatings and the corresponding target materials. In particular, in case of synthetic HA films, there is the absence of trace ions element, as expected. In the animal bone apatite coatings, sodium and magnesium ions are preserved in the coatings, without the starting precursor, confirming the possibility of transferring the composition from the target to the coating during the IJD and also in terms of ion doping. In general, the presence of both elements is much lower in the coating compared to the target, in a precursor-dependent fashion. Indeed, Mg and Na values are highest for porcine and smallest for equine bone. The lower amount of Mg and Na in the coatings can be explained by the preferential sputtering of elements (i.e., calcium) that is characteristic of plasma-assisted deposition processes, such as IJD. In spite of these differences, however, the fidelity in stoichiometry transfer can be considered high, since similar techniques result in the formation of decomposition phases.

The Ca/P and (Ca+Mg)/P molar ratios also vary between the targets and the coatings. Indeed, in the case of the Ca/P ratio, a value of 1.91 (higher than the stoichiometric one) is reported for stoichiometric apatite. For animal precursors that started from lower values, Ca/P ratios similar to HA coatings are reached, with no significant differences between the different animal sources. The (Ca+Mg)/P ratios in the coatings are found to be higher compared to those of the targets, as for the Ca/P, all data confirm a preferential sputtering of calcium.

### 3.4. The Effect of Thermal Treatments

**Figure 5:**
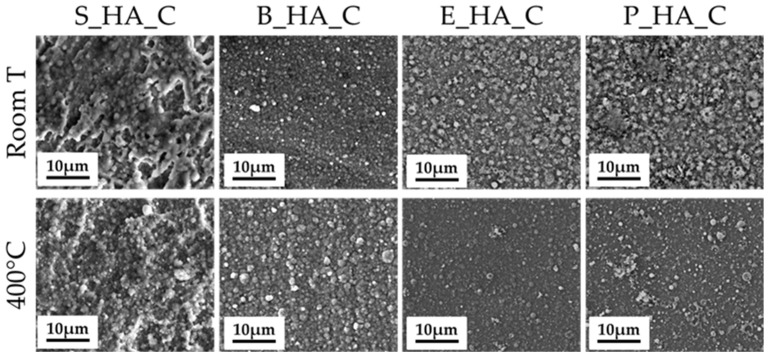
FEG-SEM images of coatings realized at room temperature with and without post-deposition annealing treatment at 400 °C (5000× magnification).

FEG-SEM investigation shows that post-treatment heating on the synthetic and naturally derived coatings does not significantly change the morphology of the films, nor does it cause any damage to the coatings (Figure 5). Indeed, no cracks, delamination or surface defects are observed.

### 3.5. Stability Profile

**Figure 6:**
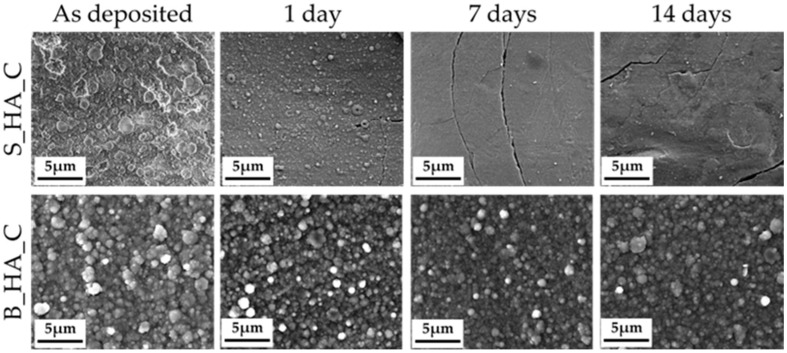
The morphology of the synthetic (S_HA_c) and biogenic coatings after 1, 7 and 14 days of immersion in α-MEM. In the figure, images of bovine bone are shown.

Film images obtained after the dissolution tests are shown in Figure 6. In particular, the dissolution tests are performed on synthetic HA coatings and compared to single naturally derived HA coatings. FEG-SEM images show that, in case of S_HA_c, the progressive immersion of HA in the medium results in an inhomogeneous dissolution, with the formation of cracks and detachments starting from 7 days of immersion. The coating already shows significant dissolution at 24 h, with a decrease in the aggregate’s dimension and number, and a general thinning and flattening of the film. The biogenic bone-derived coatings, instead, show higher stability. Indeed, the coating progressively dissolves but is stable for over 14 days in medium.

## 4. Discussion

The obtained results show the possibility of fabricating HA nanostructured thin coatings by IJD using different sources of apatite, both synthetic and natural. All the coatings are nanostructured and homogenously coat the substrate, independently of the deposition target. No surface defects, such as delamination, cracks or uncoated areas are observed. The films are composed by globular aggregates with an average diameter around 200–300 nm but have features ranging from a few tens of nanometers up to over 1 micron and a thickness of around 100–120 nm. The surface topography is important in the regulation of cellular behavior in terms of adhesion and osteogenic differentiation [39], so the micro- and nano-sized surface features obtained by IJD are desirable as they can mimic the native bone and improve the biological response of bone tissue cells. Indeed, previous results from the research group have shown that this surface nanostructuring allows one to direct cell adhesion and morphology, ultimately leading to higher differentiation towards the osteogenic lineage [19]. Although all films are nanostructured, surface geometry depends on the target, up to a certain extent. Indeed, we observe that stoichiometric HA has a flatter shape with respect to the naturally derived HA coatings, which possess more tridimensional spherical aggregates. Naturally derived coatings also show more defined aggregates with a spherical shape and surface porosity and holes in some cases. While in the case of coatings fabricated starting from synthetic hydroxyapatite the aggregates have still presented a spherical geometry, their shape is however less defined and the aggregates tend to fuse together. In addition, some changes in grain morphology are also observed by FEG-SEM, at high magnification, between the different animal sources: the E_HA_c and B_HA_c have similar characteristics with spherical grains, while the P_HA_c grain shape is less defined. Hence, selecting the appropriate targets can be used as a route to tune surface morphology. The differences in the morphology of the coatings obtained by synthetic hydroxyapatite compared to naturally derived ones is caused by the intrinsic properties and characteristics of the starting targets used during the deposition process. The final morphology of the coating depends mainly on the composition of the target and its thermal properties (which in turn are affected by porosity). The porosity/pore size distribution, mechanical properties and surface finish of the targets can also affect the sputtering process during IJD and, consequently, the final morphology of the coatings. Here, the synthetic HA targets are obtained starting from a powder, which is compressed into disks, while the animal-derived targets are fabricated from sections of the femoral diaphysis of the animal bone, which results in differences in surface morphology, mechanical properties and porosity of the targets. Porosity also varies between different animal coatings [39], explaining the differences in aggregate size distribution/shape among different animal sources.

Together with surface morphology, composition is crucial when developing new biomaterials for bone tissue engineering. Indeed, to obtain optimal behavior, biomaterials should follow the biomimetic principle, so they need to be similar to real bone in terms of composition, Ca/P ratio, crystallinity and ion doping [19]. These properties can have effects on osseointegration and osteoinduction abilities, so chemical properties such as the Ca/P ratio and ion element doping can have a relevant role in guiding these features. Our results show that IJD permits a high fidelity in the conservation of stoichiometry from the deposition target to the coating, since both the calcium phosphate-based main phase (see FT-IR Figure 5) and ion-doping (Mg and Na, Table 1) are preserved. Consequently, by using biogenic bone as a target, biomimetic coatings can be obtained. The composition of the biological HA coatings is more similar to human bone HA compared to the synthetic coatings, with the presence of trace elements sodium and magnesium, which have a relevant role on in vivo bone metabolism and regeneration.

However, Ca/P molar ratio values are slightly higher compared to the biological ones. This behavior in Ca/P ratio during the HA deposition was also obtained using other plasma-assisted techniques. For example, with the pulsed laser deposition system, Bolbasov et al. [45] obtained a coating with a Ca/P ratio of 1.87 starting from hydroxyapatite particles; Lenis et al. [46] produced HA coatings by magnetron sputtering, with a Ca/P ratio between 1.69 and 2.55 and Rau et al. [47] obtained coatings with a CaP ratio from 1.81 to 2.20, all showing promising behavior.

Although the percentage of trace elements decreases in a precursor-dependent manner, they are maintained in the coatings. So, coatings from different animal sources show different amounts of trace ions and can be selected to obtain tailored coatings.

All the films deposited at room temperature lose the crystallinity of the precursor powder, but with post-treatment annealing at 400 °C it is possible to partially recover their degree of crystallinity. The effect of the temperature treatment is similar to other HA films produced with other plasma-assisted techniques. In particular, Rau et al. [47] investigated the physiochemical properties of carbonate hydroxyapatite films, obtained through a pulsed laser deposition technique at different deposition temperatures. The FT-IR spectra showed an increase in the degree of crystallinity of the coatings by increasing the deposition temperature. In fact, in the spectra obtained for films at a lower temperature (30–200 °C), the bands around 1100–1000 cm^−1^ appear broad and poorly defined. At a higher temperature (400–750 °C), other bands appear at 1100–1000 cm^−1^, this characteristic of a higher degree of crystallinity.

While the starting targets do significantly influence the morphology of the coatings, heat treatment does not result in significant modifications of these parameters.

When samples are heat treated, good stability is found for both S_HA_C and B_HA_C coatings, as the films are visible after over 14 days in medium. However, the two show significantly different behavior. In fact, HA coatings show a higher dissolution rate so that they are significantly resorbed after 24 h in medium. In addition, progressive dissolution also results in cracking and detachments. Instead, for bone apatite coatings, dissolution proceeds gradually, with the coatings being essentially unaltered at 14 days and undergoing the occurrence of defects.

Indeed, when we studied the biological behavior of IJD coatings made of bovine bone HA [13], data showed that they can stimulate the osteogenic differentiation of human bone marrow-derived MSC (hMSC), induce a branched shape and the production of osteocalcin, alkaline phosphatase and collagen type I with the absence of any cytotoxic effects [13]. Thus, bone apatite is an ideal candidate for bioactive coatings and the future study and comparison of natural apatite from different sources appears promising to obtain tunable coatings.

The main limitation regarding biogenic apatite coatings from animal bones lies in the regulatory aspects. In fact, although animal bone is already largely used for bone substitutes in dentistry and orthopedics, it is not yet used in the coatings market, which complicates their clinical translation. In addition, since animal bones are a natural product, they present heterogeneities in composition and geometry. Although these do not significantly affect the deposition process, nor require adjustments in deposition parameters to achieve repeatability in coating properties, some standardization in the target manufacturing process still appears to be important to achieve maximum reproducibility in the coatings.

## 5. Conclusions

In this work, we have demonstrated the possibility to manufacture biomimetic nanostructured hydroxyapatite and biogenic apatite coatings, starting from different sources. In particular, the deposition of films with synthetic and naturally derived (bovine, equine and porcine bone) HAs, by ionized jet deposition, has been systematically compared.

The use of different precursors determines coating morphology and composition. Indeed, the use of biogenic, instead of stoichiometric HA, permits one to obtain coatings containing trace ions (sodium and magnesium) that have an important biological role. The amount of ion-doping is lower compared to that found in the target and is precursor-dependent.

Morphology is also influenced by the precursor, with biogenic apatite overall resulting in more spherical aggregates, although species-dependent variability is observed. These results suggest that the use of different targets can be a promising route to tune coatings characteristics.

In terms of stability, biogenic apatite coatings show better performance compared to stoichiometric ones, which undergo cracking and detachments upon prolonged exposition in medium.

Based on the obtained results, both synthetic and natural HA nanostructured coatings appear promising for application. Indeed, from a technological and regulatory point of view, synthetic HA has to be preferred, as it allows one to obtain homogeneous cylindrical targets with replicable properties, making the deposition process simpler and more standardized. On the other hand, however, naturally derived materials appear more promising, as they allow one to have a multi-doped coating, perfectly resembling the composition of bone. In addition, they show better behavior and a higher durability in simulated medium, which is key for the proposed application. Finally, from an economic and environmental point of view, using naturally derived waste materials from the food industry, such as animal bones, results in lower costs and less environmental impact than using chemical synthesis, thus representing a further advantage for these materials.

No significant differences were observed among coatings deriving from different animal precursors, either in terms of morphology or composition. This suggests that different animal sources can be used for application. However, given the lower availability of equine bones (which are less used in the food chain and are already largely used in biomaterials), and the reduced thicknesses of porcine bones causing difficulties in deposition process (lower deposition durations are permitted), bovine bones appear as the best candidate.

Overall, our data show that new green naturally derived materials can be used as hydroxyapatite sources for biomimetic nanostructured film fabrication. In particular, naturally derived HA coatings appear as a promising development in the orthopedic field, for constructs for bone regeneration and fusion. Indeed, clinically available coatings still present some drawbacks, connected to excessive thickness (leading to cracking) and to the fact that synthetic HA does not possess a composition and crystallinity similar enough to human HA. Instead, IJD biogenic coatings exhibit characteristics and chemical properties more similar to those of native human bone mineral components than synthetic materials, submicrometric thickness and a nanostructured surface morphology, all aspects known to enhance bone regeneration.

## Figures and Tables

**Table 1:** Different properties of synthetic, natural derived and human hydroxyapatite.

Ca_10_(PO_4_)_6_(OH)_2_	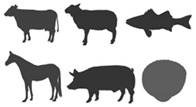	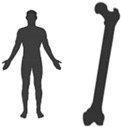
Synthetic Hydroxyapatite	Natural Derived-Hydroxyapatite	Human Bone Hydroxyapatite
Ca/P ratio 1.67	Ca/P lower than 1.67	Ca/P lower than 1.67
Absence of ion-doping	Different ion substitutions Carbonate rich	Different ion substitutions Carbonate rich
Lower solubility	Higher solubility	Higher solubility
Higher crystallinity	Lower crystallinity with respect to synthetic HA	Lower crystallinity with respect to synthetic HA

**Table 2:** The EDS elemental composition (wt.% with standard deviation s.d.) of the different targets and coating material. In particular, the Mg, Na and Ca/P molar ratio values are reported.

	S_HA		B_HA		E_HA		P_HA	
	Target	Coating	Target	Coating	Target	Coating	Target	Coating
Mg (wt.% ± s.d.)	-	-	0.57 ± 0.02	0.15 ± 0.02	0.30 ± 0.01	0.07 ± 0.04	0.52 ± 0.09	0.16 ± 0.08
Na (wt.% ± s.d.)	-	-	0.69 ± 0.44	0.21 ± 0.03	0.86 ± 0.06	0.24 ± 0.20	0.68 ± 0.01	0.53 ± 0.26
Ca/P (at.)	1.67	1.91	1.57	1.84	1.46	1.95	1.36	1.85
(Ca + Mg)/P	-	-	1.63	1.91	1.51	1.90	1.43	1.77

**Table 3:** The minimun (Dm), maximum (DM) and average (Da) diameters of the different coating aggregates (mean with standard deviation s.d.).

	S_HA_c	B_HA_c	E_HA_c	P_HA_c
Dm [nm]	79 ± 25	66 ± 35	80 ± 40	92 ± 9
DM [nm]	1372 ± 221	1329 ± 30	1328 ± 336	1432 ± 90
Da [nm]	298 ± 208	304 ± 191	304 ± 192	329 ± 215

**Table 4:** The thickness of the different coatings (mean with standard deviation s.d.).

	S_HA_c	B_HA_c	E_HA_c	P_HA_c
Room T	127 ± 11	111 ± 22	103 ± 18	119 ± 24
400 °C	122 ± 34	118 ± 31	121 ± 42	129 ± 44

## Data Availability

The data presented in this study are available on request from the corresponding authors.
